# Supplementary material for: Rotavirus VP3 targets MAVS for degradation to inhibit type III interferon expression in intestinal epithelial cells
Source: eLife. 2018 Nov 21;7:e39494. doi: 10.7554/eLife.39494 (PMC6289572; doi:10.7554/eLife.39494)
Supplement: Supplementary file 2. [file elife-39494-supp2.docx]

**Taqman and SYBR Green Primer information:**

| RV NSP5 | Probe | CY5/TCAAATGCAGTTAAGACAAATGCAGACGCT/IABRQSP |
| --- | --- | --- |
|  | Forward | CTGCTTCAAACGATCCACTCAC |
|  | Reverse | TGAATCCATAGACACGCC |
| IFNB (human) | Forward | ATGACCAACAAGTGTCTCCTCC |
|  | Reverse | GGAATCCAAGCAAGTTGTAGCTC |
| IFNL3 (human) | Forward | TAAGAGGGCCAAAGATGCCTT |
|  | Reverse | CTGGTCCAAGACATCCCCC |
| MAVS (human) | Forward | GTGCCTACTAGCATGGTGCTC |
|  | Reverse | GACCCAAGGCCCCTATTCT |
| GAPDH (human) | Forward | GGAGCGAGATCCCTCCAAAAT |
|  | Reverse | GGCTGTTGTCATACTTCTCATGG |
| Ifnl3 (mouse) | Forward | AGCTGCAGGCCTTCAAAAAG |
|  | Reverse | TGGGAGTGAATGTGGCTCAG |
| Mavs (mouse) | Taqman | Mm00523170_m1 |
| Gapdh (mouse) | Forward | TCTGGAAAGCTGTGCCGTG |
|  | Reverse | CCAGTGAGCTTCCCGTTCAG |
| Gapdh (mouse) | Taqman | Mm99999915_g1 |

**Dharmacon SMARTpool siRNA information:**

Non-targeting control: D-001810-10

AIP4 (ITCH): L-007196-00

BAZ1A: L-006941-00

BIRC6: L-013857-00

CNOT1: L-015369-01

HLTF: L-006448-00

LMO7: L-019252-00

MARCH5: L-007001-00

MAVS: L-024237-00

PHRF1: L-026727-00

PRPF19: L-004668-00

RC3H2: L-020453-02

SCAF11: L-019838-00

TNFAIP3 (A20): L-009919-00

TRIM21: L-006563-00

TRIM25: L-006585-00

TRIP12: L-007182-00

ZNF598: L-007104-00
