# Supplementary material for: Rotavirus VP3 targets MAVS for degradation to inhibit type III interferon expression in intestinal epithelial cells
Source: eLife. 2018 Nov 21;7:e39494. doi: 10.7554/eLife.39494 (PMC6289572; doi:10.7554/eLife.39494)
Supplement: Transparent reporting form [file elife-39494-transrepform.docx]

***eLife’s* transparent reporting form**

We encourage authors to provide detailed information *within their submission* to facilitate the interpretation and replication of experiments. Authors can upload supporting documentation to indicate the use of appropriate reporting guidelines for health-related research (see [EQUATOR Network](http://www.equator-network.org/%20)), life science research (see the [BioSharing Information Resource](https://biosharing.org/)), or the [ARRIVE guidelines](http://www.plosbiology.org/article/info:doi/10.1371/journal.pbio.1000412) for reporting work involving animal research. Where applicable, authors should refer to any relevant reporting standards documents in this form.

If you have any questions, please consult our Journal Policies and/or contact us: [editorial@elifesciences.org](mailto:editorial@elifesciences.org).

**Sample-size estimation**

- You should state whether an appropriate sample size was computed when the study was being designed
- You should state the statistical method of sample size computation and any required assumptions
- If no explicit power analysis was used, you should describe how you decided what sample (replicate) size (number) to use

Please outline where this information can be found within the submission (e.g., sections or figure legends), or explain why this information doesn’t apply to your submission:

The sample size and statistical analysis can be found at the end of the figure legends for each figure as well as a separate paragraph of Statistical analysis (lines 901-909) within the Materials and Methods section. The number of mice used in each in vivo experiment is indicated in the figure (Figure 5A and 5B) or provided in the figure legend (Figure 5C and Figure 5-figure supplement 1C).

**Replicates**

- You should report how often each experiment was performed
- You should include a definition of biological versus technical replication
- The data obtained should be provided and sufficient information should be provided to indicate the number of independent biological and/or technical replicates
- If you encountered any outliers, you should describe how these were handled
- Criteria for exclusion/inclusion of data should be clearly stated
- High-throughput sequence data should be uploaded before submission, with a private link for reviewers provided (these are available from both GEO and ArrayExpress)

Please outline where this information can be found within the submission (e.g., sections or figure legends), or explain why this information doesn’t apply to your submission:

All data except Figure 3C, 4E, 4F, 5A-C, and Figure 5-figure supplement 1C have been performed at least three times. Experiments in Figure 4E were performed twice. High-throughput mass spectrometry analysis (Figure 3C and Figure 4F) was performed once and the complete dataset was uploaded as Supplementary File 1. In vivo experiments (Figure 5 and Figure 5-figure supplement 1) were performed twice.

**Statistical reporting**

- Statistical analysis methods should be described and justified
- Raw data should be presented in figures whenever informative to do so (typically when N per group is less than 10)
- For each experiment, you should identify the statistical tests used, exact values of N, definitions of center, methods of multiple test correction, and dispersion and precision measures (e.g., mean, median, SD, SEM, confidence intervals; and, for the major substantive results, a measure of effect size (e.g., Pearson's r, Cohen's d)
- Report exact p-values wherever possible alongside the summary statistics and 95% confidence intervals. These should be reported for all key questions and not only when the p-value is less than 0.05.

Please outline where this information can be found within the submission (e.g., sections or figure legends), or explain why this information doesn’t apply to your submission:

Data in all bar graphs are presented as mean ± standard error of mean (SEM). Methods of statistical tests were provided at the end of figure legend for each figure and a designated paragraph of Statistical analysis (lines 901-909) in the Materials and Methods section.

(For large datasets, or papers with a very large number of statistical tests, you may upload a single table file with tests, Ns, etc., with reference to sections in the manuscript.)

**Group allocation**

- Indicate how samples were allocated into experimental groups (in the case of clinical studies, please specify allocation to treatment method); if randomization was used, please also state if restricted randomization was applied
- Indicate if masking was used during group allocation, data collection and/or data analysis

Please outline where this information can be found within the submission (e.g., sections or figure legends), or explain why this information doesn’t apply to your submission:

No randomization was applied for cell culture and in vivo experiments. Samples in Figure 3C were coded, scrambled and submitted for mass spectrometry analysis.

**Additional data files (“source data”)**

- We encourage you to upload relevant additional data files, such as numerical data that are represented as a graph in a figure, or as a summary table
- Where provided, these should be in the most useful format, and they can be uploaded as “Source data” files linked to a main figure or table
- Include model definition files including the full list of parameters used
- Include code used for data analysis (e.g., R, MatLab)
- Avoid stating that data files are “available upon request”

Please indicate the figures or tables for which source data files have been provided:

Source data for Figure 3C (MAVS immunoprecipitation-mass spectrometry counts) was provided as raw numbers as well as in the Supplemental File 1. Data for Figure 5A-C (rotavirus shedding in the stool samples in in vivo experiments) were provided as raw numbers in the format of an excel spreadsheet.
